# Combined Use of Inactivated and Oral Poliovirus Vaccines in Refugee Camps and Surrounding Communities — Kenya, December 2013

**Published:** 2014-03-21

**Authors:** Mohamed A Sheikh, Frederick Makokha, M. Abdullahi Hussein, Gedi Mohamed, Ondrej Mach, Kabir Humayun, Samuel Okiror, Leila Abrar, Orkhan Nasibov, John Burton, Ahmed Unshur, Kathleen Wannemuehler, Concepcion F. Estivariz

**Affiliations:** 1 Ministry of Health, Kenya; 2 Field Epidemiology and Laboratory Training, Ministry of Health, Kenya; 3 World Health Organization (WHO) Country Office, Nairobi, Kenya; 4 Polio Eradication, WHO, Geneva, Switzerland; 5 Inter-Country Support Team, WHO, Harare; 6 Health Section, UNICEF Kenya office; 7 United Nations High Commissioner for Refugees, Kenya; 8 Refugee Health Program, CDC/Kenya Medical Research Institute; 9 Global Immunization Division, Center for Global Health, CDC

Since the launch of the Global Polio Eradication Initiative (GPEI) in 1988, circulation of indigenous wild poliovirus (WPV) has continued without interruption in only three countries: Afghanistan, Nigeria, and Pakistan (1). During April–December 2013, a polio outbreak caused by WPV type 1 (WPV1) of Nigerian origin resulted in 217 cases in or near the Horn of Africa, including 194 cases in Somalia, 14 cases in Kenya, and nine cases in Ethiopia (all cases were reported as of March 10, 2014) (2,3). During December 14–18, 2013, Kenya conducted the first-ever campaign providing inactivated poliovirus vaccine (IPV) together with oral poliovirus vaccine (OPV) as part of its outbreak response. The campaign targeted 126,000 children aged ≤59 months who resided in Somali refugee camps and surrounding communities near the Kenya-Somalia border, where most WPV1 cases had been reported, with the aim of increasing population immunity levels to ensure interruption of any residual WPV transmission and prevent spread from potential new importations. A campaign evaluation and vaccination coverage survey demonstrated that combined administration of IPV and OPV in a mass campaign is feasible and can achieve coverage >90%, although combined IPV and OPV campaigns come at a higher cost than OPV-only campaigns and require particular attention to vaccinator training and supervision. Future operational studies could assess the impact on population immunity and the cost-effectiveness of combined IPV and OPV campaigns to accelerate interruption of poliovirus transmission during polio outbreaks and in certain areas in which WPV circulation is endemic.

During April–July 2013, a total of 14 paralytic polio cases caused by WPV1, genetically linked to a virus originating in Nigeria and also circulating in Somalia, were reported in Kenya; seven cases occurred in residents of refugee camps, six in surrounding communities, and one in a noncontiguous district but also near the Kenya-Somalia border (3) (Figure). In response to the outbreak, the Kenyan Ministry of Health conducted one national and five subnational OPV campaigns during May–November 2013. In December, the Ministry of Health administered IPV and OPV combined in a campaign directed at approximately 126,000 children aged ≤59 months including those who lived in five refugee camps (Dagahaley, Ifo 1, Ifo 2, Hagadera, and Kambioos: 98,365 children), and those in communities within five divisions that surround the camps (Dadaab, Dertu, Jarajila, Sabuli, and Liboi: approximately 27,000 children) near the border with Somalia. GPEI partners* provided funding and technical support for campaign planning and evaluation, staff training, vaccine procurement, and social mobilization. The Kenya Ministry of Health planned and implemented immunization activities with refugee camp coordinating agencies.†

## IPV/OPV Campaign Implementation

The campaign was implemented by 299 teams (173 in camps and 126 in surrounding communities) assigned to fixed (i.e., permanent) sites in health facilities and to “temporary fixed” sites in each block (in camps) or surrounding communities; mobile teams were used to reach scattered settlements of nomads. Each team included one health-care worker and two volunteers (in communities) or three volunteers (in camps). The health-care worker administered IPV (and OPV on some teams). One to two volunteers administered OPV and tallied children or marked fingers after vaccination, and one volunteer conducted door-to-door mobilization of caregivers, encouraging them to take their children to the vaccination sites. Children aged <6 weeks received OPV alone; children aged 6 weeks–59 months received OPV followed immediately by IPV.

Focus group interviews conducted before the campaign suggested ready acceptance of an injectable polio vaccine. Participants thought injections were very effective but would only accept injection by health-care workers. Caregivers also had no concerns about simultaneous administration of IPV and OPV because they viewed the two vaccines as working differently (i.e., IPV provides protection in the bloodstream, and OPV provides protection in the gut). Based on these responses, communication materials and volunteers emphasized the concept that receiving OPV is essential, but IPV can enhance immunity against polio.

## Campaign Monitoring

Vaccination activities of 47 randomly selected teams were assessed by trained campaign monitors using a standardized checklist. Of the 47 teams, 43 (91%) had sufficient staff, vaccine, and supplies to vaccinate the estimated target population, and in 39 (83%), a team member conducted door-to-door mobilization of caregivers. Of 47 vaccinators observed, five (11%) made an error in IPV administration (injection site or dosage), two (4%) prefilled syringes before the session began, and eight (17%) recapped needles during injection preparation. Errors in finger-marking or tallying of children who had received vaccine were observed in two (4%) and seven (15%) teams, respectively. Vaccines were kept in vaccine carriers with at least two ice packs at 44 (94%) of the sites. No vaccine vial monitor§ on OPV vials had a color change indicating substantial heat exposure. No vaccine vial monitors were used on IPV vials. One team was found to have frozen IPV vials; follow-up investigation revealed that these vials had been stored in a freezer before distribution to the site. Electronic temperature monitors placed inside 42 vaccine carriers during vaccination activities recorded periods of ≥60 minutes below 36.6°F (2°C) in 12 (42%) carriers and above 46.4°F (8°C) in eight (19%) carriers.

Vaccine cost was $2.09 per IPV dose¶ and $0.14 per OPV dose; the operational cost per child vaccinated during the IPV/OPV December campaign was $1.04, compared with $0.36 in the November OPV-only campaign. Estimated total cost per child vaccinated was $3.27 and $0.50 for the December and November campaigns, respectively.

No serious illnesses, hospitalizations, or deaths were reported through the passive system implemented for detecting adverse events during the week following vaccination. One child who received OPV via intramuscular injection caused by vaccinator error experienced pain and local inflammation at the injection site, and it resolved within a few days as this child was monitored.

## Coverage Survey

During December 19–23, 2013, vaccination coverage surveys were conducted using cluster survey methodology. The sampling frame was derived for camps from information provided by the United Nations High Commissioner for Refugees registry office and adapted to include areas with “unregistered” populations; campaign coordinators provided the estimated number of children aged ≤59 months for surrounding communities.** Because of the absence of a sampling frame for nomads, a convenience sample of nomadic families settled near villages participating in the survey was selected. Receipt of OPV with or without IPV in the December campaign, reasons for nonvaccination, and receipt of OPV in the November campaign were recorded for all children aged ≤59 months in each household.

Of 1,286 houses surveyed, caregiver recall information on receipt of IPV or OPV was available for 2,161 children in 1,016 households. Coverage with OPV and IPV in the December campaign was 92.8% in the refugee camps and 95.8% in surrounding communities. Receipt of OPV in the November campaign was 97.2% in the refugee camps and 97.3% in surrounding communities (Table 1).

Among 107 (5%) children aged ≥6 weeks who did not receive IPV, caregivers for 49 (46%) reported not knowing where to go for vaccination; 16 (15%) cited potential refusals (ill child, five; fear of pain, eight; and fear of adverse effects, three), and 10 (9%) children were absent during the campaign. Twelve of the 107 children who missed IPV received OPV (Table 2). Among 1,009 (99%) caregivers who were aware of the campaign, the most common sources of information were public address system or megaphone announcements (76%), a visit by a social mobilizer (47%) or health-care worker (43%), and radio (36%).

In 65 nomadic households surveyed, 40 (34%) of 118 eligible children had received IPV and OPV in the December campaign, and 37 (31%) had received OPV in the November campaign. Among children in the nomadic households, reported reasons for missing vaccine in the December campaign were lack of awareness of the campaign (70 of 76 [92%]) and not knowing where to get vaccine (six of 76 [9%]). Sources of information about the December campaign among 24 caregivers who knew about the campaign included a neighbor (54%), megaphone announcements (33%), and radio (29%).

### Discussion

Clinical trials have demonstrated that administration of IPV to children who had received OPV increases humoral and mucosal immunity to the three poliovirus serotypes more effectively than a supplementary dose of OPV (4,5). In December 2013, after a WPV1 outbreak, the Kenya Ministry of Health implemented a mass campaign with combined IPV/OPV administration in Somali refugee camps and surrounding communities in Kenya to boost population immunity levels to ensure interruption of any residual WPV transmission and prevent spread from potential new importations. This population was considered at greater risk because of the high number of cases reported in the outbreak, prior importations of WPV and vaccine-derived polioviruses (3), and frequent population movement between Somalia and major Kenya cities in the area.

Several challenges to the use of IPV in campaign settings have been noted previously, including 1) increased cost and operational complexity; 2) potentially reduced coverage, because injectable vaccines cannot be delivered house-to-house; and 3) concerns about caregiver mistrust of IPV or their rejection of OPV-only campaigns in the future. Factors that contributed to the success in overcoming these challenges for this campaign in Kenya included 1) strong commitment from the Ministry of Health and coordination among implementing partners in developing comprehensive operational plans and allocating resources quickly, 2) flexibility to move “temporary fixed” sites frequently in response to caregiver demands to bring vaccine closer to their homes, and 3) high acceptance of IPV by caregivers, as shown by the high coverage and the small proportion of unvaccinated children (15%) who missed vaccine because of potential refusals. Vaccination coverage in a subsequent OPV-only campaign conducted in February 2014 in the area was similar to coverage in previous campaigns, showing that IPV use in one campaign did not negatively impact a subsequent OPV campaign.

Challenges in field implementation of the IPV/OPV campaign can provide lessons for future campaigns. Observation of vaccinators revealed errors in injection technique and in IPV use; similar findings have been identified with other injectable vaccines used in campaign settings (6,7), stressing the need for appropriate training of vaccinators and supervisors. Both vaccines are recommended to be stored and transported at 36.6°F–46.4°F (2°C–8°C), but this study found that temperatures inside vaccine carriers might be above or below the recommended range. Whereas OPV would not be affected by low temperatures, and vaccine vial monitors would indicate when vaccine has been damaged by heat, IPV vials used in this campaign did not have vaccine vial monitors, and staff members were unaware that IPV can be damaged by freezing (8). Comprehensive precampaign planning of cold chain requirements, consideration of vaccine vial monitor inclusion on IPV vials, and appropriate staff training on existing guidelines for prevention of vaccine damage from heat or freezing (8) will be important to prevent loss of vaccine effectiveness in future campaigns. Additionally, a survey in nomadic settlements found low campaign awareness and a high proportion of children who did not receive vaccine during either the November or December campaigns, suggesting that certain settlements are missed repeatedly. Additional strategies, including improved communications, are needed to track and access nomadic populations during all vaccination campaigns and reflect seasonality of nomadic movements.

As part of the Polio Eradication and Endgame Strategic Plan 2013–2018,†† which aims to discontinue all use of OPV after eradication of WPV, IPV is to be introduced by the end of 2015 into the routine immunization schedules of 126 countries that use only OPV (9,10). The Kenya experience has shown that IPV also can be provided in campaigns with high coverage and community acceptance, although at a higher cost than OPV-only campaigns and requiring particular attention to training and supervision. IPV/OPV campaigns could be considered to improve population immunity and accelerate interruption of poliovirus transmission in other polio outbreaks and in certain areas where WPV transmission is endemic. Operational studies during future campaigns should assess the impact on population immunity and the cost-effectiveness of this strategy in different settings.

What is already known on this topic?

Results from clinical trials have suggested that administration of inactivated poliovirus vaccine (IPV) in combination with oral poliovirus vaccine (OPV) through mass campaigns in certain settings could achieve the high population immunity levels required to interrupt poliovirus transmission with fewer campaigns. IPV, administered by intramuscular injection, has not been used in campaigns because of concerns about the increased cost and operational complexity, potential reduction in coverage, and potentially lower caregiver acceptance.

What is added by this report?

The first community-based IPV/OPV campaign was conducted during December 14–18, 2013, in Kenya in response to a wild poliovirus type 1 outbreak. The campaign targeted an estimated 126,000 children aged ≤59 months who lived in five refugee camps and in communities surrounding the camps in five divisions near the Kenya-Somalia border. A survey estimated coverage with both vaccines at 92.8% in refugee camps and 95.8% in surrounding communities.

What are the implications for public health practice?

The Kenya experience has shown that combined IPV/OPV campaigns are feasible and can achieve high coverage and community acceptance. Future IPV use in campaigns might consider the following: 1) conducting population-specific studies to guide social mobilization and delivery strategies, 2) assessing cold chain needs before and during the campaign, 3) allocating vaccination teams with skilled staff members and clear work duties to minimize errors, 4) addressing injection technique and cold chain during training of vaccinators and supervisors, and 5) using specific strategies to reach nomadic and other hard-to-reach populations.

## Figures and Tables

**FIGURE:**
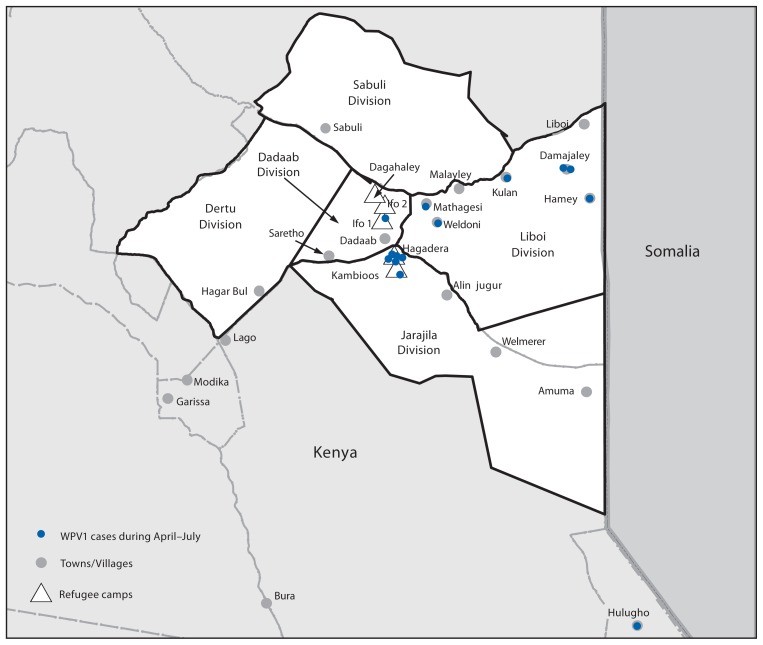
Five divisions targeted during a combined IPV/OPV vaccination campaign in refugee camps and surrounding towns/villages — Kenya, December 2013 **Abbreviations:** IPV = inactivated poliovirus vaccine; OPV = oral poliovirus vaccine; WPV1 = wild poliovirus type 1.

**TABLE 1:** Vaccination coverage with inactivated poliovirus vaccine (IPV) and oral poliovirus vaccine (OPV) during a December campaign and with OPV only during a November campaign,* by refugee camp and surrounding communities† — Kenya, 2013

Study area	Target population size	Percentage who received both IPV and OPV in December campaign			Percentage who received OPV only in November campaign		
	
		No. surveyed	(%)	(95% CI)	No. surveyed	(%)	(95% CI)
Dagahaley camp	23,815	299	(83.3)	(73.5–89.9)	301	(95.7)	(91.4–97.9)
Ifo 1 camp	22,350	270	(94.1)	(89.6–96.7)	238	(92.9)	(85.2–96.7)
Ifo 2 camp	21,560	331	(98.2)	(95.6–99.3)	331	(99.4)	(97.8–99.8)
Hagadera camp	24,660	340	(95.3)	(90.5–97.7)	338	(99.4)	(96.5–99.9)
Kambioos camp	5,980	328	(96.3)	(90.3–98.7)	326	(100.0)	—
Total camps	98,365	1,568	(92.8)	(90.2–94.8)	1,534	(97.2)	(95.4–98.3)
Surrounding communities	21,831	593	(95.8)	(93.5–97.3)	590	(97.3)	(95.0–98.5)
**Overall**	**120,196**	**2,161**	**93.3**	**(91.2–95.0)**	**2,124**	**(97.2)**	**(95.4–98.3)**

**Abbreviation:** CI = confidence interval.

* Infants aged <6 weeks received OPV only. Children aged 6 weeks–59 months received OPV followed by IPV. Receipt of vaccination was documented by caregiver.

† Residents of communities in the following divisions: Dadaab, Dertu, Jarajila, and Sabuli; Liboi Division was excluded from the survey for security reasons.

**TABLE 2:** Reasons reported by caregivers for children aged ≥6 weeks not receiving inactivated poliovirus vaccine during a December vaccination campaign in refugee camps and surrounding communities*— Kenya, 2013

Reasons	Children in refugee camps (n = 90)		Children in surrounding communities (n = 17)		Overall (N = 107)	
		
	No.	(%)	No.	(%)	No.	(%)
**Communication/Social mobilization**						
Unaware of campaign	7	(8)	0	—	**7**	**(7)**
Didn’t know where to get vaccine	47	(52)	2	(12)	**49**	**(46)**
**Delivery issues**						
Vaccination site too far	3	(3)	0	—	**3**	**(3)**
Vaccination time inconvenient	4	(4)	0	—	**4**	**(4)**
**Individual reasons**						
Ill child	5	(6)	0	—	**5**	**(5)**
Fear of pain from injection	3	(3)	5	(29)	**8**	**(7)**
Fear of adverse effects from vaccine	2	(2)	1	(6)	**3**	**(3)**
Child absent during vaccination activities	6	(7)	4	(24)	**10**	**(9)**
Reason not recorded	13	(14)	5	(29)	**18**	**(17)**

* Twelve children received oral poliovirus vaccine only; 95 did not receive either vaccine.

## Footnotes

* The GPEI initiative was founded in 1988 with the following spearheading agencies: the World Health Organization, Rotary International, CDC, and UNICEF; the Bill and Melinda Gates Foundation subsequently became a major supporter of GPEI.

† The organizations coordinating health services in refugee camps are Medecins Sans Frontieres in Dagahaley, Islamic Relief Kenya in Ifo 1, Kenya Red Cross in Ifo 2, and the International Rescue Committee in Hagadera and Kambioos. The United Nations High Commissioner for Refugees oversaw implementation in all camps.

§ OPV can be frozen without having impact but potency is affected by high temperatures, which can be detected by vaccine vial monitors on the label. The vaccine vial monitor changes color when a vial has been exposed to excessive temperature over time that has likely damaged the vaccine. Vaccine can be used if the inner square is lighter than the outer circle but must be discarded if the inner square is the same color or darker than the outer circle. IPV is sensitive to freezing and heat and recommended to be stored and transported at 36.6°F–46.4°F (2°C–8°C); IPV vials used in this campaign had no vaccine vial monitors.

¶ The IPV cost included vaccine, syringes, needles, and shipment. The OPV cost included vaccine and shipment.

** Eligible households were those in which children aged ≤59 months resided. Liboi Division was excluded from the survey because of insecurity.

†† Additional information available at http://www.polioeradication.org/resourcelibrary/strategyandwork.aspx.
